# The use of beta-tricalcium phosphate bone graft substitute in dorsally plated, comminuted distal radius fractures

**DOI:** 10.1186/1749-799X-6-24

**Published:** 2011-05-22

**Authors:** Michael G Jakubietz, Joerg G Gruenert, Rafael G Jakubietz

**Affiliations:** 1 Department of Trauma-, Hand-, Plastic and Reconstructive Surgery, University of Wuerzburg, Wuerzburg, Germany; 2 Department of Hand, Plastic and Reconstructive Surgery, Kantonspital St. Gallen, Switzerland

## Abstract

**Background:**

Intraarticular distal radius fractures can be treated with many methods. While internal fixation with angle stable implants has become increasingly popular, the use of bone graft substitutes has also been recommended to address comminution zones and thus increase stability. Whether a combination of both methods will improve clinical outcomes was the purpose of the study

**Methods:**

The study was thus conducted as a prospective randomized clinical trial. 39 patients with unilateral, intraarticular fractures of the distal radius were included and randomized to 2 groups, one being treated with internal fixation only, while the second group received an additional bone graft substitute.

**Results:**

There was no statistical significance between both groups in functional and radiological results. The occurrence of complications did also not show statistical significance.

**Conclusions:**

No advantage of additional granular bone graft substitutes could be seen in this study. Granular bone graft substitutes do not seem to provide extra stability if dorsal angle stable implants are used. Dorsal plates have considerable complication rates such as extensor tendon ruptures and development of CRPS.

## 

Fractures of the distal radius are the most common fractures in the upper extremity and treatment options have been controversially discussed throughout the literature. Closed reduction is almost always easy to achieve but is difficult to maintain, resulting in a loss of reduction. Therefore, treatment aims to prevent radial shortening, malunion, and articular incongruity as these factors are associated with poor outcomes [1]. Treatment varies from splinting and minimally invasive percutaneous pinning to open reduction with external or internal fixation [2]. Internal fixation can be done through a volar, dorsal or combined approach. While volar fixed angle implants could be the future for treatment of most Colles' fractures, the dorsal approach remains a good choice in highly comminuted fractures with a metaphyseal defect, and when a bone graft is also required [2]. Open reduction of dorsally dislocated fractures is often done through a dorsal approach because of the advantages it offers: fracture reduction under direct vision with the possibility of dorsal capsulotomy to directly visualize the articular surface and small fragments. It also offers the possibility to repair associated intercarpal injuries through the same approach [3]. Furthermore this approach allows for easy bone augmentation. Bone grafting is usually recommended in such cases to provide structural support and thus to prevent radial shortening and loss of radial height [4,5]. In terms of bone grafting several options exist. Autogenous bone grafts from the iliac crest are the best choice, but have the disadvantage of donor site morbidity such as vascular and nerve injury, iliac wing fractures and infection besides adding operative time and costs [6,7]. Allograft bone grafts have the inherent risk of infection and thus some surgeons are reluctant to use them [8]. The focus of research has been on the development of bone graft substitutes. Several artificial bone graft substitutes are available which imitate cancellous bone grafts: calcium phosphates, calcium sulfates and coralline hydroxyaptatites. Osteoinductive and osteoconductive properties are claimed by many manufacturers while even the definition of these terms is vague, as Amy Ladd has pointed out [8]. Although these materials have gained wide spread popularity, there is no clear proof of its effects in combination with internal fixation of radius fractures [9-12]. Most randomized studies on bone graft substitutes included different treatment regimens, thus the sole effect of bone graft substitutes cannot be clearly estimated [13,14]. To this date no study has clearly shown the effect of bone graft substitutes when internal, angle stable fixation is used in patients over the age of 50, where osteoporosis may be present. The aim of this study was to evaluate effects of a bone graft substitute in such circumstances. Granular beta-tricalcium phosphate was chosen due to the texture of the material, which allows filling of the comminution zone more easily than solid substances, which need to be broken into pieces, first. The study was thus conducted as a prospective randomized clinical trial. One group was treated with a bone graft substitute in addition to internal fixation of the radius fracture, whereas the second group was treated with internal fixation only. The only difference was the use of the bone graft substitute, while all other treatment modalities were similar.

## Materials and methods

Thirty-nine patients with unilateral, intraarticular fractures of the distal radius were included. All patients gave informed consent and permission of the institutional ethical committee was obtained. Inclusion criteria were age over 50, fractures of AO-type C with a dorsal comminution zone and at least two instability criteria. Open fractures were excluded as well as additional osseoligamentous injuries of the extremity, such as carpal injuries. Fractures were classified, using plain radiographs, into subgroups of C I - C III after the AO - System. Patients were randomized to either group I (20 patients), which received a dorsal implant only (Pi-Plate, Synthes Corporation), or group II (19 patients) which, additionally to the implant, received bone augmentation with granular beta-tricalcium phosphate (Chronos, Synthes Corporation). Surgery was carried out according to the techniques described previously. The defects in group II were filled with the granular phosphate after internal fixation was completed. Granular material had been chosen due to the possibility to fill the defect after reposition. With the implant in place, the defect was filled with the granules, which were compressed into the dead space with a dasher. We had previously found that method to be more effective than blocks or wedges which could often only be inadequately fitted to the shape of the defect. To prevent accidental placement of granules into the joint, visual control of the joint and irrigation were done after completion of augmentation procedure. Arthrotomy was carried out in all patients to estimate intraarticular steps and confirm the reposition afterwards. Great care was also taken to prevent tendon irritations by using retinacular flaps to protect the extensor tendons. Furthermore all patients underwent an additional posterior interosseus nerve neurotomy. Postoperative treatment consisted of 2 weeks cast immobilization followed by another 4 weeks immobilization in a removable splint accompanied by motion exercises. Weight bearing, resistive exercises were started 6 weeks postoperatively. All examinations were performed by a handsurgeon other than the primary surgeon, but for reasons of patient satisfaction, the primary surgeon saw the patient on every visit as well. Results were evaluated 6 weeks, 3, 6 and 12 months postoperatively focusing on functional recovery and radiographic outcome. Functional measurements evaluated wrist flexion and extension, pronation and supination as well as ulnar and radial abduction. Grip strength was measured using JAMAR dynamometer and compared to the opposite side. A neurological examination was also carried out at every visit. Radiological evaluation included frontal and lateral standard views. Articular surface, intraarticular steps, height of the radius, radial inclination, ulnar variance and palmar tilt were measured. All implants were removed 6 months postoperatively. DASH and Gartland scores were evaluated 12 months postoperatively. The categoric variables were analysed using SPSS^® ^(SPSS GmbH Software, Munich, Germany, Version 11.5.1) software. After explorative analysis, the Student-T test was used except in 2 occasions were the Mann-Whitney test was applied when the Kolmogorov-Smirnov test showed that non-parametric variables were not distributed normally.A two-sided p-value < 0.05 was considered statistically significant.

## Results

39 consecutive patients were included. The mean age was 67.7 years in group I and 67.3 in group II. In group I 85% were females, 15% males, in group II 84.2% females and 15.8% males. Fractures were classified using the AO classification. In group I 45% (9p) were C1 fractures, 25% (5p) C2 fractures and 30% (6p) C3 fractures, whereas in group II 42.1% (8p) were C1, 21.1 (4p) C2 and 36.8 (7p) C3 fractures. Both groups displayed a normal variance in terms of fracture classification. The preoperative dorsal tilt was 34 degrees in group I versus 27 degrees in group II, radial inclination 11 versus 14 degrees, radial height 7 versus 8 mm, and ulnar variance 4.6 versus 5.2 mm. In group I, 65% (13p) showed a fracture of the ulnar styloid (73.7% (14p) in group II). In no case osteosynthesis was required. 2 patients in group II had acute median nerve compression and were treated with carpal tunnel release. No infections and fracture nonunions occurred. Functional outcomes were evaluated at 1.5, 3, 6 and 12 months (Table 1). After one year grip strength averaged 70% of the opposite side in the augmented group and 75% in the nonaugmented. Active range of motion was increased in the nonaugmented group in comparison to the augmented group. Combined active flexion and extension compared to the opposite side were 65 versus 56%, radial and ulnar duction 75 versus 68%, while combined pronation and supination were 87 versus 76%. No statistical significance could be found between the groups. Pain levels decreased continuously over the observation period in both groups and also did not display statistical significance (p = 0.858). Hardware was removed 6.7 months (range 5-12) in the nonaugmented and 6.2 months (range 3-8) postoperatively in the augmented group. All fractures showed bony union after 12 weeks. Radiological measures were taken at 1.5, 3, 6 and 12 months postoperatively (Table 2). Again, there was no statistical significance between the groups. The volar tilt was 13.37 degrees in group 1 after 12 months and 14.18 in group 2 (p = 0.690). Radial inclination measured 22.5 degrees in group 1 and 23.7 in group 2 respectively (p = 0.455). Radial height was 12 mm in group 1 and 12.7 mm in group 2 (p = 0.369), while ulnar variance was 2 mm and 2.9 mm respectively (0.132). Beginning degenerative, posttraumatic osteoarthrosis (Grade II in the Knirk Jupiter Grading system) had developed in one patient out of each group, while grade I was seen in 9 patients of each group. Complications such as secondary displacement of a fragment and intraarticular steps greater than 2 mm occurred in a total of 7 patients (3 in group I, 4 in group II). In five patients the dislocation required secondary osteosynthesis with a fixed angle volar plate. Developing CRPS was diagnosed in 8 patients and successfully treated with cortisone (3 Group I, 5 Group II), while no case of complete manifestation of CRPS was observed. All cases of CRPS were seen after the initial surgery, none was seen after hardware removal. Extensor tendon ruptures occurred in 3 patients as ruptures of the index finger EDC II and required operative treatment (2 in group I, 1 in group II). The DASH1 score was 14.26, DASH2 27.99 in group I, 21.72 and 39.58 in group II, with no statistical significance between the groups. The Gartland score was similar in both groups [10].

**Table 1:** Functional results

	6 weeks			3 months			6 months			12 months		
	**n**	**a**	**p-value**	**n**	**a**	**p-value**	**n**	**a**	**p-value**	**n**	**a**	**p-value**

Flexion	21.6	19.2	0.368	30.5	29.4	0.786	37.8	31.3	0.112	46.8	37.3	0.089

Extension	20.5	21.1	0.808	24.3	28.5	0.188	33.9	37.6	0.332	44.3	39.0	0.527

Radial abduction	8.5	8.9	0.976	13.3	13.2	0.805	15.4	16.3	0.581	17.1	15.7	0.874

Ulnar abduction	18.8	17.4	0.676	25.3	23.5	0.549	31.2	30.5	0.932	30.2	24.1	0.370

pronation	66.0	66.3	0.919	63.5	64.5	0.744	75.6	77.9	0.165	78.3	71.0	0.115

supination	21.8	28.8	0.226	43.8	46.7	0.556	62.1	63.7	0.454	62.9	58.0	0.345

N = nonaugmented, A = augmented

**Table 2:** Radiological results

	6 weeks			3 months			6 months			12 months		
	**n**	**a**	**p-value**	**n**	**a**	**p-value**	**n**	**a**	**p-value**	**n**	**a**	**p-value**

Volar tilt	15.1	14.8	0.734	14.3	15.2	0.723	13.3	14.0	0.747	13.4	14.2	0.690

Radial inclination	20.7	22.1	0.344	21.1	22.5	0.491	21.5	22.7	0.463	22.5	23.7	0.455

Radial height	10.8	11.7	0.364	11.4	11.8	0.771	11.9	12.4	0.679	12.0	12.8	0.369

Ulnar variance	1.0	1.8	0.228	1.7	2.4	0.551	2.0	2.7	0.609	2.0	2.9	0.132

N = nonaugmented, A = augmented

## Discussion

Intraarticular fractures of the distal radius are challenging to treat. The abundance of treatment options shows that to this date no perfect solution for all fracture types does exist. There is no consensus as which method or combination should be employed in severe fractures, with multiple techniques popularized throughout the hand surgery community [13-22]. Severe fractures are treated operatively by most. While a shift from dorsal to volar plates has occurred, no randomized studies exist to this date to show a clear advantage of the volar approach in severe fractures. After an initial euphoria about palmar plating systems in the most severe fractures the majority of hand surgeons has learned that dorsal plating still has its role. Especially high-grade intraarticular fractures with significant dorsal comminution zones are difficult to treat. An established option is dorsal plating with Pi-Plates, which offer angle stability when additional pins are used together with screws [3]. The Pi-Plate has never become widely popular due to tendon irritations and the need for hardware removal. To this date no plating system, either dorsal or volar can offer fixation without the risk of tendon irritations [3,15,17]. Even rounded heads of minimally protruding palmar screws have shown to irritate and ultimately rupture extensor tendons. Tendon irritations are inherent sequelae of the dorsal approach, regardless of the system used. The dorsal surface of the distal radius and its close proximity to the extensor tendons with absent muscle coverage leave little space for a plate. The use of smaller, less prominent plates has decreased the risk, but not completely eliminated it. Even advanced dorsal plates by Rikli have been shown to cause tendon ruptures [15]. Our own experience with a large case number of dorsal plates has shown a reasonable risk when hardware is removed in all patients. For these reasons all hardware was removed 6 months postoperatively. Nevertheless several tendon ruptures have occurred in our patients. Other complications such as development of CRPS have also been described before [3]. While no statistical significant conclusion can be presented, the authors feel that this may be triggered by the mere existence of hardware in the dorsal compartment, which leads to irritation, inflammation and ultimately development of CRPS. No cases of CRPS were diagnosed after hardware removal. Bone grafting has been widely advocated in severe fractures to fill metaphyseal defects. Long before angle stable fixation was available, surgeons had to employ artistic techniques of several plates and often adding cortical iliac crest grafts to achieve stability [18]. Interest in bone graft substitutes stems from added morbidity and cost associated with iliac crest bone grafts and potential transmission of infectious diseases in allograft materials. Especially calcium phosphate derivates have been in the focus of research [8]. Most randomized studies on bone graft substitutes included different treatment regimes, thus the effect of bone graft substitutes cannot be clearly estimated [16]. Furthermore, most authors include fracture patterns from A2-C3 fractures, which rather shows the variability of the methods than the specific use of the bone graft substitutes. This study was designed that the only difference in the subgroups was the use of the bone graft substitute. We found no statistically significant difference between the groups postoperatively. The bone-augmented group showed neither improved clinical nor radiological outcomes. Volar and radial inclination, ulnar variance and radial height were similar to the group without bone substitute. Secondary dislocations were also evenly distributed among the groups with 4 in the augmented group and 3 in the non-augmented group, all occurred in AO-type C III fractures. Secondary dislocations are the result of massive comminution which prevents stable fixation of the fragments and was also not influenced by additional bone grafts. Filling of the dead space of the comminution zone can only marginally increase stability of the construct, and thus decreasing the risk of secondary dislocation. A shortcoming of the study could be the use of granular bone graft substitutes. It remains unclear if the use of solid substitutes might have added stability, although in contrast to corrective osteotomy the shape of the defect cannot be addressed with a single block. In our experience it proved impossible to completely fill defects with solid materials only, therefore it remains speculative if solid materials would prevent secondary dislocation despite the theoretical advantage they offer. Augmentation materials should rather provide volume to fill the defect and thus triggering osteo-in- and -conductivity, than merely providing punctual structural support. We also believe that secondary dislocation seen in this study is a sequela of the fixation of extremely comminuted fractures rather than of the augmented material, since no central impressions of the articular surface were seen, but volarly displaced fragments. Fragments require screw fixation, while bone augmentation mainly supports the central articular surface. Another aspect is a possible weakness of the material after hardware removal. In case of incomplete integration additional loss of height and/or inclination could become obvious. The augmented group did not show a significant difference to the nonaugmented group after removal of the hardware. After 6 months the material seemed to be integrated and the bone remodeled, as neither positive nor negative aspects, such as loss of angulation and height could be observed in the further observation period. There are other limitations to our study. The cohort of patients was collected at a tertiary care center with expertise in dorsal plating systems. It is not known whether these results can be generalised to all patients, as there is definitely a referral-bias in our patient population. Also our inclusion criteria were deliberately strict to limit our patient population to elderly patients with low energy trauma where plate fixation may be problematic. These patients require stable fixation for poor bone quality to allow quick rehabilitation. This again may increase complication rates compared to other studies.

As noted by other authors stable fixation of the fracture is the most important factor for good healing [13,19-24]. Nonunions did not occur in any case. Bone healing is rarely a problem in older patients, since the osteopenic metaphyseal cancellous bone heals readily due to the relatively increased blood supply [8]. Increased osteogenic or osteoinductive properties of the augmentation material were thus not observed in the augmented group, a fact which cannot be generalized. In this study additional granular bone augmentation showed no advantage over pi-plate fixation alone. The results cannot be generalized to all types of angle stable implants and bone graft substitutes, therefore no conclusion about other angle stable implants or substitutes can be drawn. The results show that a recommendation for general use of bone graft substitutes cannot be made, these products should be rather confined to certain situations, such as considerable bone loss in high energy trauma. Angle stable fixation is the key component in regard to restoring and preserving anatomical position. The importance of angle stable fixation is further proven by the fact that volar, angle-stable fixation does not require bone augmentation. With further development of angle stable implants, either volar or dorsal plates, it remains doubtful if this type of bone grafting will have substantial effects on the outcome of distal radius fractures in the future.

## Competing interests

This study was in part financially supported by IBRA.

None of the authors has any conflict of interest in terms of commercial or financial involvement. No agreement with IBRA was made regarding the prohibition of publishing positive or negative results.

## Authors' contributions

MJ drafted the manuscript, was involved in the design of the study, did the statistical interpretation and analysis. JG carried out the examinations, was involved in the development of the study. RJ developed the design of the study, carried out the examinations. All authors performed the surgeries. All authors read and approved the final manuscript.
